# Study on Shear Creep Characteristics of the Discontinuities with Different 3D Morphologies

**DOI:** 10.3390/ma16010405

**Published:** 2023-01-01

**Authors:** Qingzhao Zhang, Zejun Luo, Ying Chen, Zhen Wang

**Affiliations:** 1 Department of Geotechnical Engineering, Tongji University, Shanghai 200092, China; 2 School of Business Administration, Shanghai Lixin University of Accounting and Finance, Shanghai 201620, China; 3 School of Mechanical Engineering, Nanjing University of Science and Technology, Nanjing 210094, China

**Keywords:** natural rock mass discontinuity, 3D morphology characteristics, creep test, damage mechanics, constitutive model

## Abstract

The rheological phenomenon of rock mass affects the long-term safety of rock mass engineering. In this study, gneiss samples with different 3D morphologies are prepared by splitting tests and are tested through multi-step creep tests. The long-term strength of rock discontinuities is determined by using several methods. The test results show that as the 3D morphological parameter increases, the creep deformation, creep rate, and the duration of failure all decrease. The long-term strength of rock discontinuities is linearly related to the 3D morphological parameter. Based on the principle of damage mechanics for rock mass, a damage variable is introduced in the creep model, and an improved non-linear Burgers model is established. Research results are of great theoretical significance and practical value for the design, construction, and long-term safety of rock mass engineering.

## 1. Introduction

The discontinuity of rock mass, as the weak structural planes in rock mass, plays a controlling role in the mechanical properties of rock mass [1]. The existence of discontinuities complicates the engineering properties of rock mass, which is the key to anisotropy and heterogeneity of rock mass. In recent years, infrastructure construction in West China has developed rapidly, with longer and deeper tunnels passing through active fault zones with high ground stress appearing, such as Ya’an-Linzhi Tunnel of Sichuan-Tibet Railway, and Erlangshan Tunnel of Sichuan-Tibet Highway. The instability and failure of deep tunnels often starts from the deterioration of rock discontinuities. Under natural conditions, rock discontinuities will not fail within a short period of time; the long-term mechanical properties of rock mass are the most important factors affecting the stability and safety of engineering [2,3]. Therefore, the study of the creep characteristics of rock mass discontinuities is of great value.

Experimental study is an important and indispensable means to study the creep characteristics of rock mass. At the beginning of the last century, the study of creep behavior started and many researchers have published works on the creep characteristics of rock and rock discontinuities in the past decades [4,5,6,7,8,9,10]. From these research results, the creep characteristics of rock discontinuities are similar to those of intact rocks. For example, the three typical creep stages (i.e., transition creep stage, steady creep stage, and accelerated creep stage) can also be observed in the shear creep curve of rock discontinuities. The creep rate depends on the ratio of shear stress to shear strength [11,12]. In addition, there is also a threshold stress regarded as the long-term strength during the creep process of rock discontinuities. Some researchers have focused on the factors influencing the creep characteristics of rock discontinuities. Asanov and Pan’kov [13], performed shear creep tests of rock discontinuities with different normal stress conditions. Zhang et al. [8] investigated the creep behavior of white marbles with weak structural planes under four normal stress conditions, and adopted a non-linear flow component to improve the Burgers model based on the testing results.

Different from intact rocks, the complex surface properties of rock discontinuities control the mechanical behavior of rock discontinuities. Previous research studies have shown that the morphological properties of rock discontinuities are one of the important factors which remarkably affect the strength and deformation characteristics of rock discontinuities under creep conditions [14,15,16,17]. The rock joint roughness coefficient (JRC) proposed by Barton and Choubey [18] has been recommended by the International Society for Rock Mechanics and Engineering to evaluate morphological characteristics of rock discontinuities. Zhang et al. [19] and Wang et al. [10] prepared samples of artificial discontinuities based on JRC and analyzed the impact of discontinuity roughness on creep characteristics. In recent years, the development of surface measurement technology has introduced new methods to describe 3D morphological properties of rock discontinuities with new morphological parameters [20,21,22]. However, the investigations related to the shear creep testing of rock continuities with 3D morphologies are limited.

**Figure 1:**
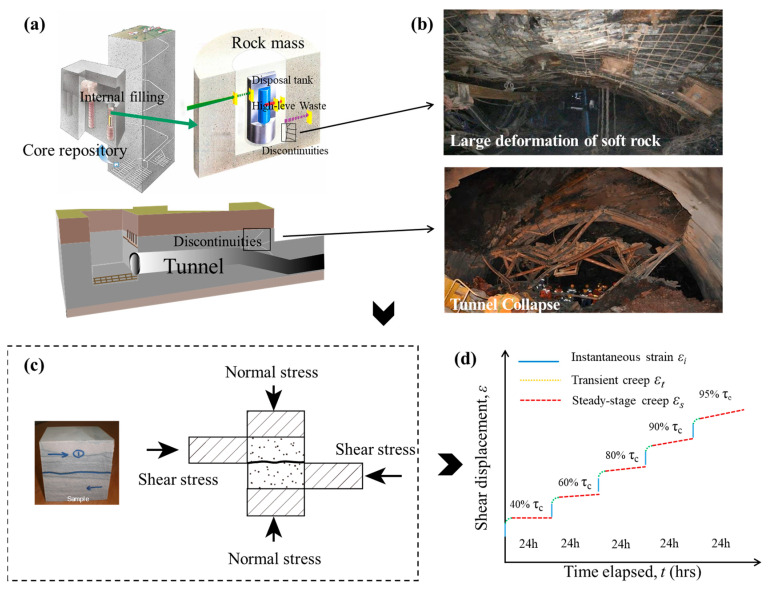
Creep test of rock discontinuities: (**a**) Rock mass discontinuities in underground rock mass engineering; (**b**) Large deformation of soft rock and tunnel collapse; (**c**) Schematic diagram of shear creep test; (**d**) Shear creep curve of rock mass discontinuities.

In this study, based on the 3D roughness evaluation method for discontinuities, multi-step creep tests are conducted on natural rock discontinuities formed by splitting (Figure 1). The creep characteristics of discontinuities with different 3D morphologies are investigated in detail. A creep model with a damage variable is established, describing the creep damage characteristics well.

## 2. Materials and Methods

### 2.1. Sample Preparation

**Figure 2:**
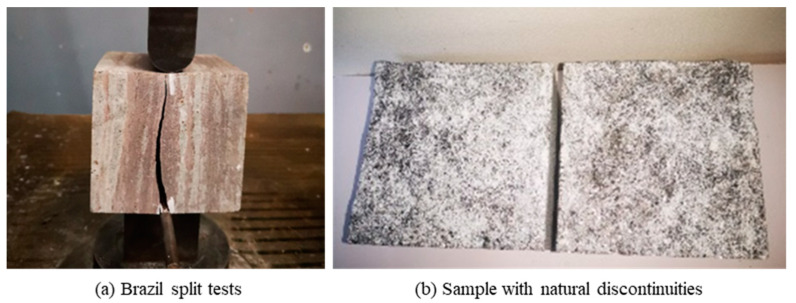
Sample preparation for shear creep tests: (**a**) Brazil split tests; (**b**) Sample with natural discontinuities.

**Figure 3:**
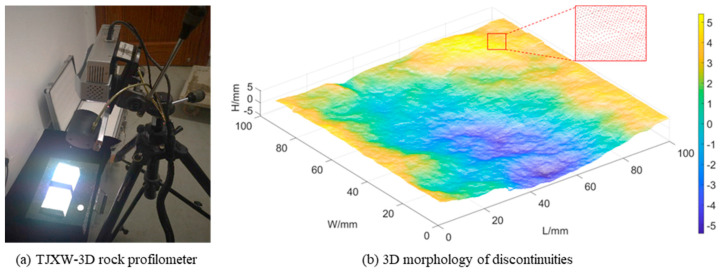
3D morphological evaluation of rock mass discontinuities: (**a**) TIXW-3D rock profilometer; (**b**) 3D morphology of discontinuities.

All the samples for this test were obtained from natural gneiss blocks with 100 mm × 100 mm × 100 mm (L × W × H). In order to simulate the natural rock mass discontinuities, the discontinuities were obtained by Brazil split tests in the direction parallel to that of the gneiss schistosity, as shown in Figure 2. Before the shear creep tests, the surface morphology of rock mass discontinuities was measured by a TJXW-3D rock profilometer (Figure 3).

As Zhang et al. [23] stated, the effective shear resistance of the discontinuities is mainly determined by the parameters facing the shear direction. The root-mean-square ($Z_{2}$) is proved to be a parameter which can better describe the morphological characteristics of discontinuities [24,25]. In this study, the root-mean-square ($Z_{2}$) of relief slope considering the shear direction of discontinuities was selected to quantitatively evaluate the 3D morphological characteristics of discontinuities. According to the effective shear dip ($\theta^{*}$) proposed by Grasselli et al. [21], Zhang et al. [23] have proposed a formula of calculating $Z_{2}$ as shown in Equation (1):

(1) $$Z_{2}=\sqrt{\frac{\sum_{i=1}^{n}\left(\tan^{2}\theta_{i}^{*}\right)}{n}}$$

where *n* refers to the number of effective shear dips on the whole rock discontinuities surface. The morphological characteristics parameter *Z*_2_ takes the shear direction of rock mass discontinuity into consideration, and the anisotropy of the discontinuity is considered in the evaluation process. The samples with different $Z_{2}$ of discontinuities were used in the next shear creep test, and the root-mean-square values of the discontinuities are given in Table 1.

### 2.2. Test Equipment and Procedures

**Figure 4:**
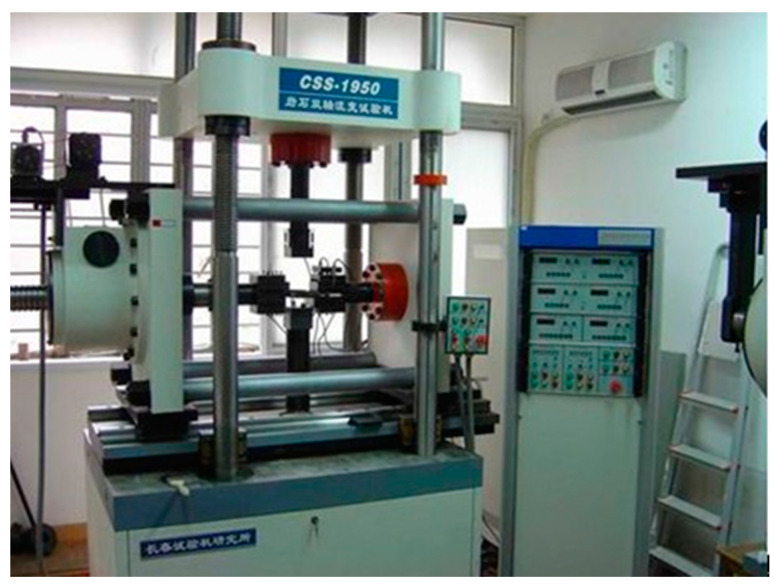
Biaxial rheological testing system (CSS-1950).

The shear creep tests of discontinuities were carried out on a servo-controlled biaxial rheological testing machine (CSS-1950) in Tongji University (Figure 4). The maximum compressive loads in the vertical and horizontal directions were 500 kN and 300 kN, respectively. The accuracies of the loading control system and the deformation measurement were less than 1% and 0.5%, respectively.

**Figure 5:**
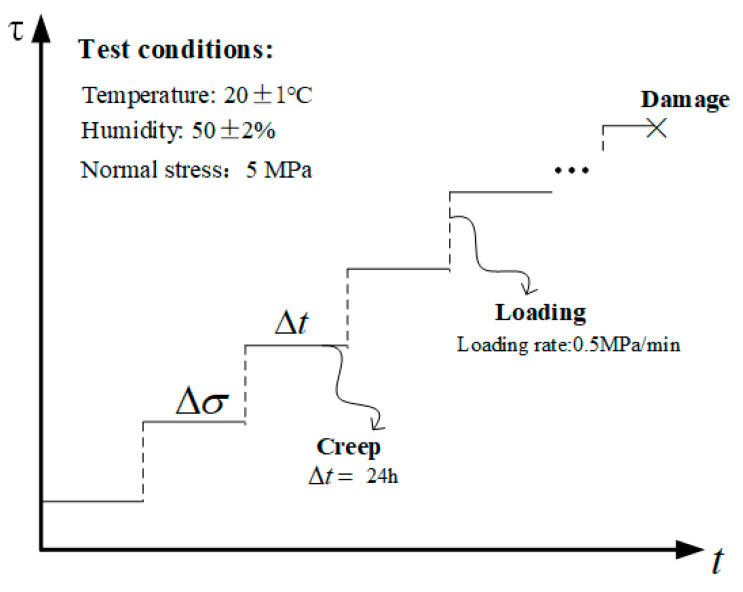
Shear stress path of the shear creep test.

The basic mechanical parameters of rock mass discontinuities are determined through relevant basic mechanical tests. The average uniaxial compressive strength ($\sigma_{c}$) was obtained as 50.5 MPa through the uniaxial ultimate compressive strength test. The average shear strength obtained by the direct shear test under a normal stress of 5 MPa (0.1$\sigma_{c}$) was 4.32 MPa. The shear creep tests are used to study the rheological characteristics of the discontinuities. Considering the design size of the testing machine, the size of the discontinuities is 100 mm × 100 mm × 100 mm (L × W × H). In this study, the normal stress of 5 MPa was applied and the shear rate maintained a constant rate of 0.5 MPa/min until the sample failed. The samples were sheared consecutively under six shear stress levels which are approximately 50%, 60%, 70%, 80%, 90%, and 100% of the shear strength (4.32 MPa), as shown in the Table 1. The duration of each stress level was set 24 h. If the sample did not damage at 100% of the shear strength, the shear stress would be increased to a higher level with the same increment until the sample damaged. The shear stresses and the last stress level are listed in Table 1. During the whole test process, the temperature was controlled at 20 ± 1 °C, and the humidity was controlled at 50 ± 2% to minimize any effects of temperature and moisture fluctuations on the test results. Figure 5 shows the relationship between shear stress and time under the shear creep test.

## 3. Shear Creep Characteristics of Rock Discontinuities

### 3.1. Creep Deformation of Different 3D Morphological Discontinuities

**Figure 6:**
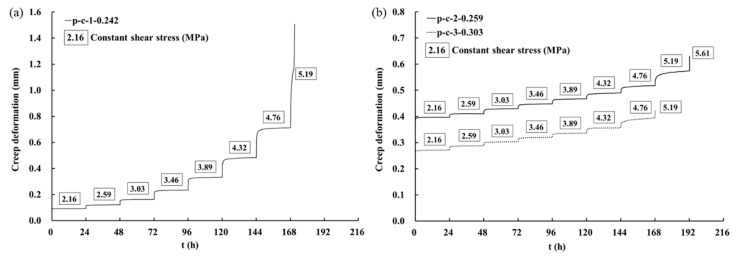
Creep curves of discontinuities with three types of surface morphology: (**a**) p-c-1; (**b**) p-c-2 and p-c-3.

Figure 6 shows the creep curves for the whole test process of the discontinuities with three types of surface morphology. The creep deformation accumulated with time and increased obviously at the higher stress level stages. When the shear stress level reached the last stage, the sample failed quickly. It can be found that under the same creep stress, the creep deformation was influenced by the morphological parameter of discontinuities ($Z_{2}$). The characteristics of the creep curves were similar in all tests, but the total deformation of p-c-1 was significantly greater than that of p-c-2 and p-c-3. The sample in p-c-1 had the smallest $Z_{2}$, and its total deformation reached 1.6 mm (Figure 6a). The result shows that under the same shear stress, the creep deformation and the total deformation decreased with the increase of $Z_{2}$ of the discontinuities.

**Figure 7:**
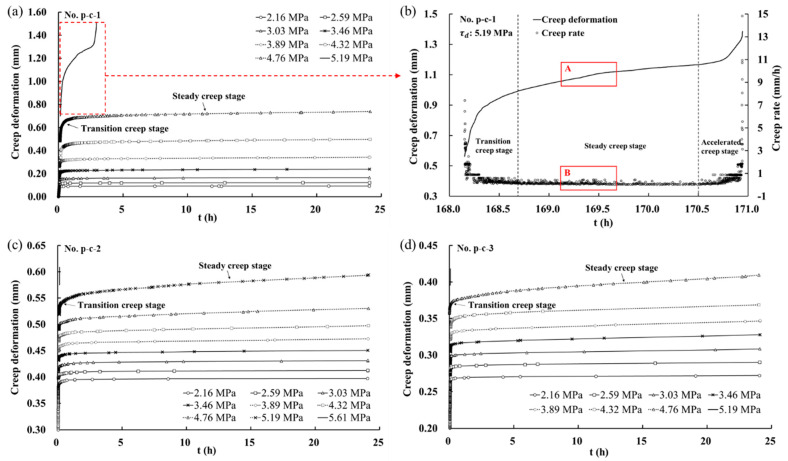
Grading creep curves of discontinuities with three types of surface morphology: (**a**) p-c-1; (**b**) creep curve and creep rate curve ($\tau_{d}$ = 5.19 MPa of p-c-1); (**c**) p-c-2; (**d**) p-c-3.

Figure 7 shows the grading creep curves of the discontinuities under various shear stress levels. The creep deformation increased slightly when the stress level was low at previous levels, while the creep deformation significantly increased under high stress levels, as shown in Figure 7a,c,d. Similar to the characteristics of the creep curve of rock, the shear creep curves of the discontinuities can be divided into three creep stages, i.e., transition creep stage, steady creep stage, and accelerated creep stage. Actually, due to the uncertainty of failure time, it was difficult to observe the three creep stages in the creep curve of the last stress level in many tests. In this study, only the creep curve at the last stress level of p-c-1 exhibited three creep stages (Figure 7b), while the samples in p-c-2 and p-c-3 failed rapidly at the last stress level. The creep curves at the last stress level of p-c-2 and p-c-3 can be considered to show that the accelerated creep stage occurred directly and the samples failed rapidly due to the absence of the transition creep stage and the steady creep stage.

The creep curve of p-c-1 at the last stress level was selected and the relationship between creep deformation and creep rate was shown in Figure 7b. The transition creep stage was from the beginning of creep to 168.7 h, then the creep went into the steady creep stage and remained in this stage until 170.5 h, and finally went into the accelerated creep stage until damaged at 170.9 h. In the steady creep stage, the creep rate changes slightly, and the creep curve appears to be approximately linear. However, the creep curve may fluctuate with time and show a local rise or decline, as shown in Zone A in Figure 7b. Accordingly, the creep rate fluctuated clearly in Zone B which corresponded to the creep curve in Zone A. The reason should be that the shear crack and asperity crushing induced the change of the resistance between discontinuities, resulting in the fluctuation of the creep rate.

The surface morphology of the rock discontinuities affects the creep characteristics. As $Z_{2}$ increased, the duration of the accelerated creep stage became shorter and the sample failed faster. This could be explained as that the creep process made a transition from friction to cutting on the discontinuities, leading to more severe failure in the accelerated creep stage.

### 3.2. Creep Rate of Different 3D Morphological Discontinuities

**Figure 8:**
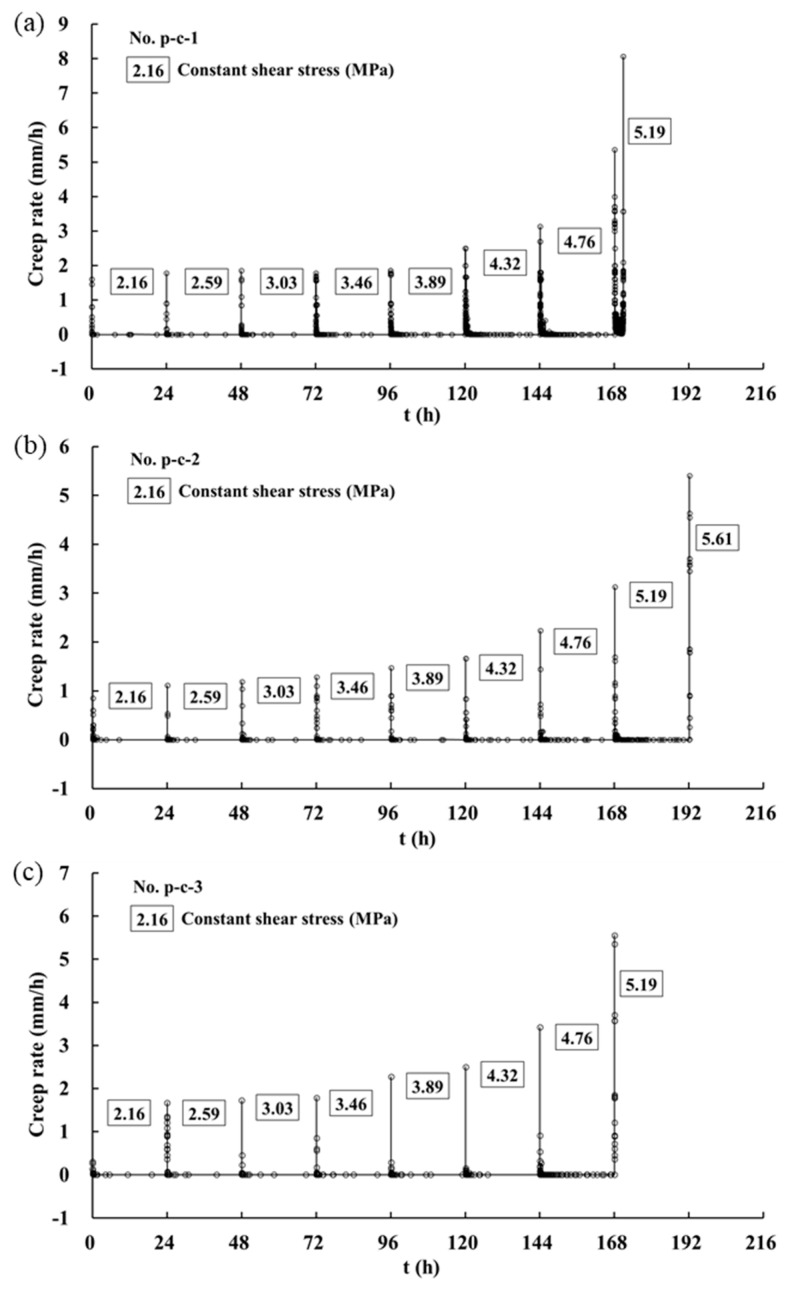
Creep rate curves of discontinuities with three types of surface morphology: (**a**) p-c-1; (**b**) p-c-2; (**c**) p-c-3.

Figure 8 shows the creep rate of the whole process under the normal stress of 5 MPa, where the creep rate is the ratio of creep deformation increment to time increment (*v_D_* = ΔD/Δt). The creep rate slowly increased as the shear stress increased in previous loading steps, and when the shear stress level exceeded a certain value, the creep rate increased significantly. For example, the initial creep rate of p-c-1 was almost constant in the previous five shear stress levels and then increased suddenly when the shear stress was 4.32 MPa (Figure 8a). The initial shear creep rate under the shear stress of 4.32 MPa was 1.68 times higher than that when the shear stress was 3.89 MPa. For the samples of p-c-2 and p-c-3, the initial shear creep rate displayed an abrupt increase when the shear stress was 3.89 MPa, as shown in Figure 8b,c.

**Figure 9:**
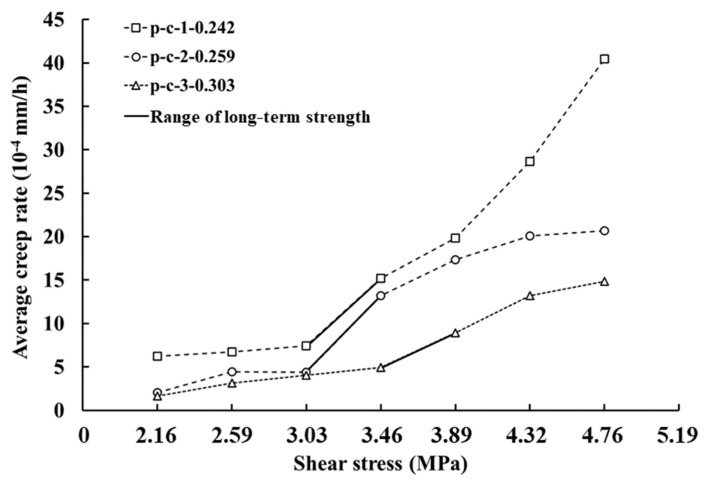
Creep rate behavior of discontinuities with three types of surface morphology in steady creep stage.

According to the analysis of the whole-process curve characteristics, the discontinuities showed steady creep until 8–24 h after loading, where the creep rate was approximately linear. Thus, the steady creep rate was taken from the average of creep rate within 8–24 h, regarded as average creep rate. Figure 9 shows the relationships between the average creep rate and shear stress in the shear creep tests. It can be observed that as the 3D morphological parameter ($Z_{2}$) increased, the average creep rate of each shear stress level decreased. In addition, there was a threshold stress level in all shear creep tests. Taking p-c-1 as an example, when the shear stress was lower than 3.03 MPa, the average creep rates basically remained at the same level, which was 6.25 × 10^−4^, 6.74 × 10^−4^ and 7.40 × 10^−4^ mm/h, respectively. However, when the shear stress surpassed 3.03 MPa, the average creep rate increased greatly from 7.4 × 10^−4^ to 15.2 × 10^−4^ mm/h, as the shear stress increased from 3.03 to 3.46 MPa. Similarly, the average creep rates of p-c-2 and p-c-3 also showed a substantial increase if the shear stress exceeded a critical stress level. Therefore, it can be preliminarily determined that the critical value of p-c-1 and p-c-2 may range from 3.03 MPa to 3.46 MPa, while that of p-c-3 may range from 3.46 MPa to 3.89 MPa. The reason should be related to the expansion of cracks or non-linear growth of the viscoplastic strain rate in the creep process [26].

## 4. Long-Term Strength of Rock Discontinuities

In general, the creep deformation consists of instantaneous, viscoelastic, and viscoplastic deformation during the whole creep process. The viscoplastic deformation is the primary cause for the failure of the sample, because it can result in the deterioration of mechanical properties of the discontinuities. In shear creep tests, the elastic deformation accounts for a dominant part of the deformation under lower stress, and the curve appears to be approximately linear. When the stress is higher than a certain “threshold stress,” the viscoplastic deformation rate increases significantly, and the shear curve becomes non-linear [7,27]. Meanwhile, the creep deformation and creep rate increase dramatically. Hence, the sudden change of creep rate can also be used as one of the criteria for determining the long-term strength [28]. The estimated range of the long-term strength can be obtained from Figure 8 and listed in Table 2.

**Figure 10:**
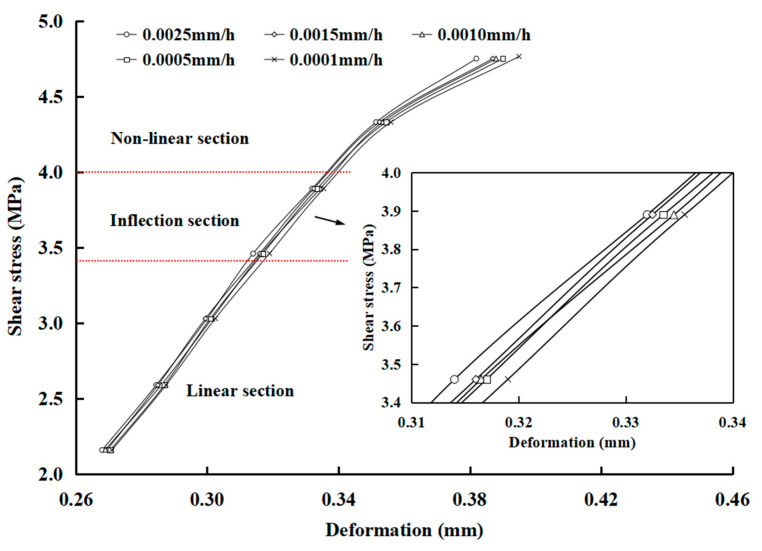
Iso-creep rate curves of p-c-3.

Actually, the steady creep stage and accelerated creep stage appear only when the stress is greater than the long-term strength. According to the inflection point method proposed by Wang et al. [12], each time point of the creep curve corresponds to a unique deformation and creep rate under a shear stress level. As a result, when a specific point in time is determined, the corresponding creep deformation, creep rate, and stress levels can be obtained. Finally, the iso-creep rate curves can be drawn in the stress–deformation coordinate system, as shown in Figure 10. Taking the sample of p-c-3 as an example, all the curves can be divided into linear and non-linear sections by an inflection point (Figure 10). When the stress is less than 3.5 MPa, the curves show a linear relationship, and then become non-linear with an inflection point when the shear stress is higher than 3.5 MPa. The inflection point is considered as a threshold at which viscoplastic deformation accounts for a dominant part of creep deformation. When the stress is higher than that at the inflection point and continues to increase, the viscoplastic deformation will increase greatly, and the non-linear feature of the curves is more obvious. Therefore, the stress value corresponding to the inflection point can be determined as the long-term strength in the shear creep tests of rock discontinuities.

**Figure 11:**
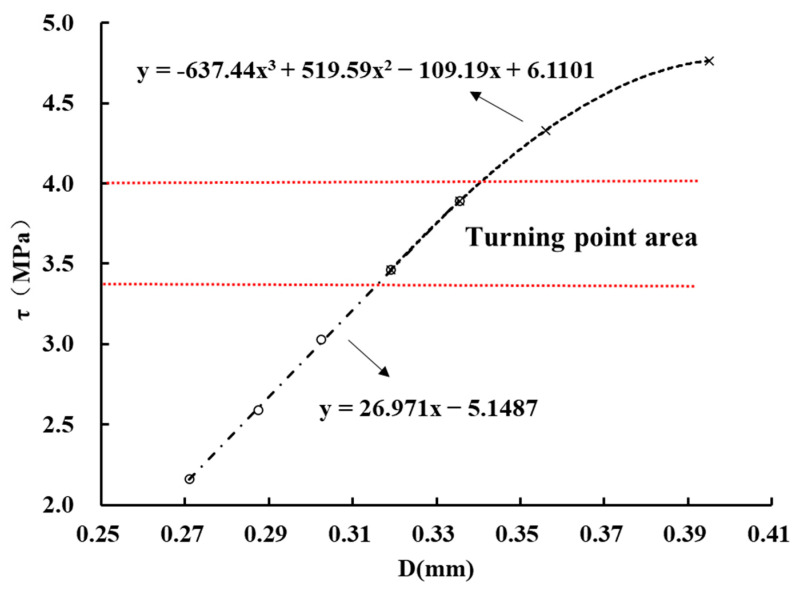
Determination of inflection point in the iso-creep rate curve of p-c-3 by Levenberg–Marquardt method.

**Figure 12:**
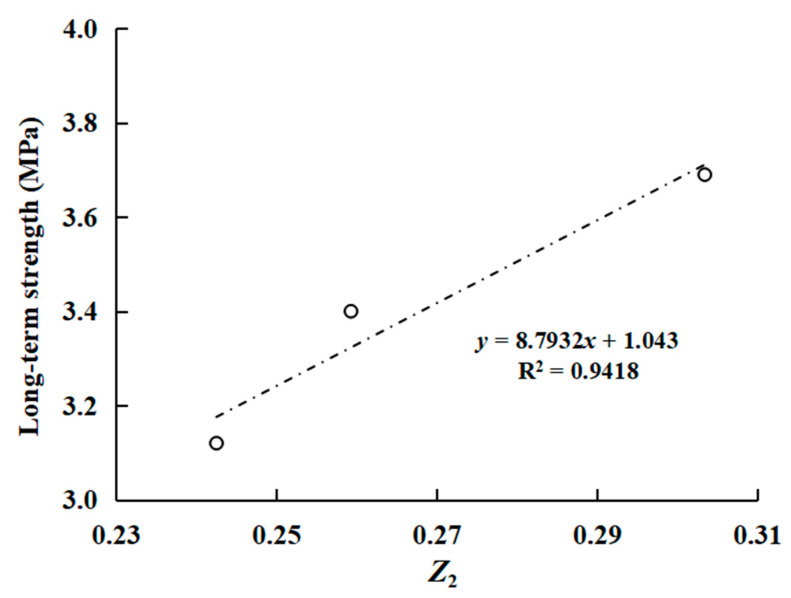
Relationship between long-term strength and $Z_{2}$ of the discontinuities.

Based on the previous analysis, the determination of inflection points is critical to obtaining the long-term strength. According to the Levenberg–Marquardt algorithm method [29], the linear section and non-linear section of the iso-creep rate curve were fitted, respectively, as shown in Figure 11. The inflection point was determined by the minimum slope difference between the linear section and non-linear section at the inflection point. In the same way, the inflection points of other iso-creep rate curves can be determined and the long-term strength is the average value of the inflection points of five iso-creep rate curves. Thus, the long-term strengths of three samples were obtained by the iso-creep rate curve method and listed in Table 2. It can be found that the long-term strengths obtained by the transition creep method and the inflection point method are consistent, and the inflection point method has better reliability in determining long-term strength. Figure 12 shows the relationship between the long-term strength and the 3D morphological parameter ($Z_{2}$). The long-term strength increases with the increase of $Z_{2}$, and shows a linear trend.

## 5. Non-Linear Burgers Model Based on Damage Mechanics

**Figure 13:**
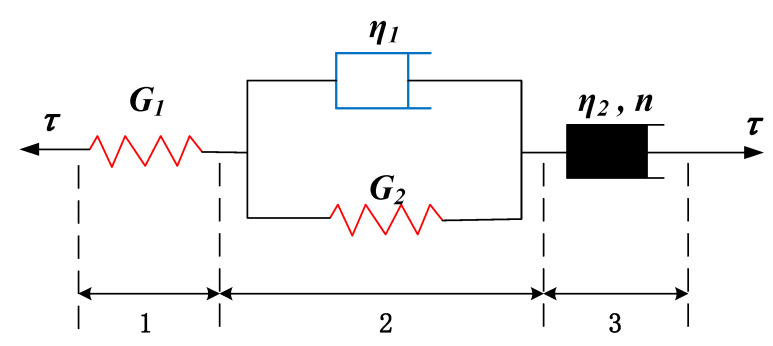
Non-linear Burgers model (modified from Zhang et al. [8]).

Resulting from the characteristics of the creep curves in this study, an improved model considering damage mechanics can be proposed based on the non-linear Burgers model proposed by Zhang et al. [8]. Figure 13 shows the non-linear Burgers model, the third part of which is the non-linear flow component. According to the non-linear Burgers model and initial condition, the creep equation from the rheological constitutive equation can be obtained as follows:

(2) $$\mu =\tau_{0}\left\{\frac{1}{G_{1}}+\frac{1}{G_{2}}\left[1-exp\left(-\frac{G_{2}}{\eta_{1}}t\right)\right]+\frac{t^{n}}{\eta_{2}}\right\}$$

where *G*_1_ and *G*_2_ refer to shear modulus, *η*_1_ and *η*_2_ refer to viscosity coefficients that reflect the creep rate of discontinuities, and *n* refers to rheological index.

The creep process is inevitably accompanied by damage, which is dependent on the time. Based on the variation law of damage variable, the damage variable (*D*) in the time interval from creep initiation to failure was used to represent the damage during the creep process. The expression of the damage variable can be assumed as follows:

(3) $$D=1-e^{-bt}$$

where *b* refers to a constant related to the discontinuity material, which is greater than 0. When *t* = 0, *D* = 0; when *t* = *t*_b_, *D* = 1, of which *t*_b_ refers to the time that the sample is damaged. Based on the non-linear Burgers model, the damage variable was introduced to express the stress of the second part (see Figure 12), that is, τ was replaced by $\tau /\left(1-D\right)$ in the equation under the initial condition that *t* = 0 and $\mu_{2}=0$. Therefore, the creep equation considering damage can be written as:

(4) $$\mu =\tau_{0}\left[\frac{1}{G_{1}}+\frac{1}{G_{2}+b\eta_{1}}\left(e^{bt}-e^{-\frac{G_{2}}{\eta_{1}}t}\right)+\frac{t^{n}}{\eta_{2}}\right]$$

**Figure 14:**
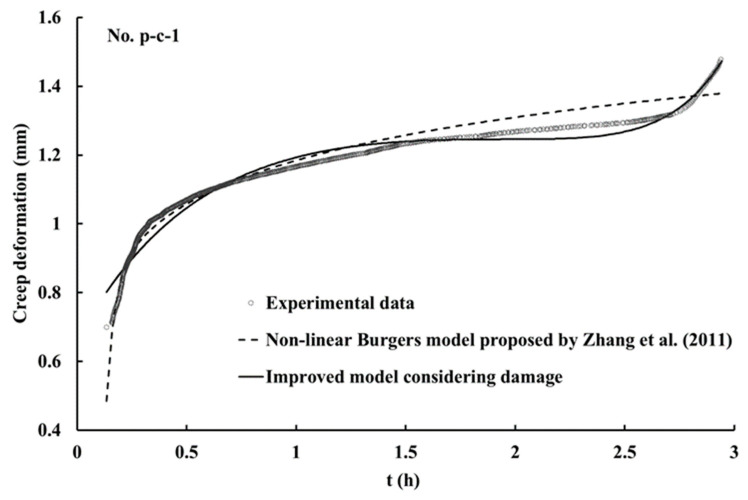
Fitting curves of p-c-1 at the last stress level (Partial model from Zhang et al. [8]).

Compared with the previous non-linear Burgers model, the improved model considers the change in the damages of discontinuities in the creep process, and gives a description of the effective shear modulus of discontinuities in the creep process through time function, so as to achieve a fine simulation of the accelerated creep state of discontinuities. Based on Equation (4), the creep test data are fitted to obtain the fitting parameters of the non-linear Burgers model based on damage mechanics in Table 3. These parameters can be used to obtain fitting curves of the discontinuities, as shown in Figure 14. From the fitting parameters and curves, the improved model shows a high fitting degree with the experimental data, especially in the accelerated creep stage. The value of n obtained by model calculation is 11.744 at the last loading stress, which is greater than 1, indicating that the fitting curve shows an accelerated creep stage; when n is less than 1, the creep curves of discontinuities maintain in the steady creep stage. The model results are the same as the test results. The non-linear Burgers model based on damage mechanics can reflect the creep process of discontinuities better in details, and describe the accelerated creep stage better.

## 6. Conclusions

In this paper, a series of shear creep tests of rock discontinuities was carried out to study the effect of 3D morphological features on the shear creep characteristics of rock discontinuities. Research results are of great theoretical significance and practical value for the design, construction, and long-term safety of rock mass engineering. The conclusions are summarized as follows:

(1) For the same normal stress, the 3D morphological features of discontinuities have a great influence on the shear creep characteristics. As the 3D morphological parameter increases, the duration of failure decreases and the damage is more intense. The creep deformation and creep rate decreased with increasing $Z_{2}$.
(2) The long-term strength range of discontinuities determined by transient creep method and the long-term strength value further determined by inflection point method are in good agreement. The long-term strength increases linearly with the increase of $Z_{2}$.
(3) Based on the non-linear Burgers model, a damage variable of discontinuities is employed, and an improved model considering damage mechanics is further proposed. This model can describe the accelerated creep characteristics of discontinuities and fits well with the experimental data under each stress level.

## Figures and Tables

**Table 1:** Parameters of rock mass discontinuities under shear creep test.

Sample No.	$Z_{2}$	$\sigma_{n}$ (MPa)	$\tau_{l}$ (MPa)
p-c-1	0.242	5	2.16, 2.59, 3.03, 3.46, 3.89, 4.32, 4.76, 5.19
p-c-2	0.259	5	2.16, 2.59, 3.03, 3.46, 3.89, 4.32, 4.76, 5.19, 5.61
p-c-3	0.303	5	2.16, 2.59, 3.03, 3.46, 3.89, 4.32, 4.76, 5.19

**Table 2:** Long-term strengths of the discontinuities determined by different methods.

Sample No.	Morphological Parameter	Transient Creep Method/MPa	Inflection Point Method/MPa
p-c-1	0.243	3.03~3.46	3.12
p-c-2	0.259	3.03~3.46	3.4
p-c-3	0.303	3.46~3.89	3.69

**Table 3:** Fitting parameters of non-linear Burgers model based on damage mechanics.

Sample No.	τ/MPa	G_1_/(MPa/mm)	G_2_/(MPa/mm)	b	$\eta_{1}$/(MPa·h)	$\eta_{2}$/(MPa·h)	n	R
p-c-1	2.16	24.443	203.276	0.00232	6.474	−140.811	−0.291	0.885
	2.59	23.552	3.832	0.00027	0.060	−3.874	−0.001	0.942
	3.03	19.705	3.005	−0.36330	819.631	−3.31^10^	−7.575	0.974
	3.46	20.410	1.976	0.00011	0.047	−2.039	−0.002	0.979
	3.89	15.894	1.420	0.00006	0.038	−1.460	−0.002	0.991
	4.32	4.350	1.189	−9.05921	4.188	−6.954	−0.077	0.985
	4.76	49.553	0.612	−0.00040	0.019	−0.661	−0.005	0.993
	5.19	8.222	0.418	−1.68975	4.450	6.3^6^	11.744	0.987
p-c-2	2.16	5.393	106.157	−2.49633	3.294	−1478.965	−1.349	0.896
	2.59	65.231	1.050	−20.08536	21.215	7.136	0.006	0.963
	3.03	47.233	1.016	−15.49373	22.152	8.585	0.009	0.961
	3.46	58.000	0.925	−11.48285	33.941	9.211	0.007	0.963
	3.89	48.541	0.904	0.00003	0.014	−0.992	−0.001	0.991
	4.32	44.475	0.745	−0.38736	−609.753	10.990	0.020	0.974
	4.76	50.612	0.804	0.00009	0.015	−0.864	−0.001	0.996
	5.19	36.964	0.741	−0.46771	−353.883	12.261	0.043	0.991
p-c-3	2.16	7.742	216.871	−1.16286	9.658	−167.460	−0.249	0.872
	2.59	45.544	1.478	0.00009	0.017	−1.700	0.000	0.989
	3.03	46.956	1.444	0.00014	0.020	−1.627	0.000	0.985
	3.46	48.637	1.256	0.00014	0.019	−1.379	0.000	0.991
	3.89	57.757	1.235	0.00014	0.021	−1.349	0.000	0.997
	4.32	53.657	1.079	0.00008	0.019	−1.157	−0.001	0.996
	4.76	59.646	1.029	−0.00534	5340.550	15.986	0.012	0.998

## Data Availability

All data, models, and code generated or used during the study appear in the submitted article.
